# Endoplasmic reticulum stress-induced PCD and caspase-like activities involved

**DOI:** 10.3389/fpls.2014.00041

**Published:** 2014-02-14

**Authors:** Yao-Min Cai, Jia Yu, Patrick Gallois

**Affiliations:** Faculty of Life Sciences, University of ManchesterManchester, UK

**Keywords:** programmed cell death, VPE, proteasome, UPR, plant caspases

## Abstract

Plant cells, like cells from other kingdoms, have the ability to self-destruct in a genetically controlled manner. This process is defined as Programmed cell death (PCD). PCD can be triggered by various stimuli in plants including by endoplasmic reticulum (ER) stress. Research in the past two decades discovered that disruption of protein homeostasis in the ER could cause ER stress, which when prolonged/unresolved leads cells into PCD. ER stress-induced PCD is part of several plant processes, for instance, drought and heat stress have been found to elicit ER stress-induced PCD. Despite the importance of ER stress-induced PCD in plants, its regulation remains largely unknown, when compared with its counterpart in animal cells. In mammalian cells, several pro-apoptotic proteases called caspases were found to play a crucial role in ER stress-induced PCD. Over the past decade, several key proteases with caspase-like enzymatic activity have been discovered in plants and implicated in PCD regulation. This review covers what is known about caspase-like enzymatic activities during plant ER stress-induced PCD and discusses possible regulation pathways leading to the activation of relevant proteases in plants.

## INTRODUCTION

Plant cells have the ability to self-destruct in a controlled manner. This process is called programmed cell death (PCD). Unlike death as a consequence of physical damage, which is not controlled by the cell itself, PCD is genetically regulated. PCD is a versatile tool for plants to cope with various situations or needs. In development, tracheary elements for example, undergo PCD to form the xylem (Groover et al., 1997; Bollhöner et al., 2012). PCD also is used in plant defense. When plant leaves are attacked by pathogens, PCD can be elicited locally where the tissue is invaded. In this case, PCD contributes to reducing the growth of the pathogen and possibly its spreading into other parts of the plant (Greenberg, 1996). Among the various stresses that induce plant PCD, endoplasmic reticulum (ER) stress is a new comer and is gaining more and more attention. The ER is an essential organelle. One of its functions is folding and *N*-glycosylation of secreted proteins. If for various reasons these processes do not function properly, misfolded proteins will accumulate inside the ER and cause ER stress. Cells experiencing ER stress will try to restore ER homeostasis by inducing the expression of unfolded-protein-response (UPR) genes. The products of these genes help cells escape ER stress by increasing the protein folding capacity and secretion ability within the ER. However, if the mounted rescue response is overwhelmed by ER stress, cells will eventually enter PCD. Such an ER stress-induced PCD has been observed in plants when *Arabidopsis* seedlings were treated with increasing concentration of PEG8000, which mimics drought. ER stress was induced and eventually resulted in PCD of root cells (Duan et al., 2010). In addition, when plants are challenged with biotic stress, for example a pathogen, ER stress is observed (Moreno et al., 2012) and this may contribute to the induction of PCD. In the past few years, there has been several reports on how ER stress regulators regulate UPR genes in plant cells (Liu and Howell, 2010; Howell, 2013). However, we still have a limited picture of the PCD pathway components activated when ER stress is unresolved. Among PCD regulators, caspases are central components that mediate animal PCD pathways in response to various stimuli, including ER stress. Caspases are cysteine-dependent aspartate specific proteases. As the name suggests, caspases cleave their substrates after an aspartate residue (in P1 position in the cleavage site). This led to the development of synthetic tetra-peptides conjugated with fluorescent reporters to determine caspase enzymatic activities. For example, Tyr(Y)-Val(V)-Ala(A)-Asp(D) is used to measure caspase-1 activity because it is the four amino acid pattern preferentially cleaved by caspase-1. Of note, YVAD can also be cleaved *in vitro* by other caspases or unrelated proteases such as legumains (Rotari et al., 2001; McStay et al., 2007). In plants, enzymatic assays using these synthetic caspase substrates have directly or indirectly implicated proteases with caspase-like activities in ER stress-induced PCD (Zuppini et al., 2004; Costa et al., 2008). The term caspase-like is used because there is no caspase ortholog in plant genomes and the plant proteases closest to animal caspases, the metacaspases, are unable to cleave synthetic caspase substrates and have instead Arg specificity. The plant caspase activities must therefore originate from proteases unrelated to animal caspases. Using various experimental systems, several research groups were able to identify plant proteases that possessed caspase-like enzymatic activities and regulated PCD. Plant proteases related to caspases and proteases with caspase-like activities have been extensively reviewed (Van der Hoorn, 2008; Xu and Zhang, 2009; Tsiatsiani et al., 2011; Vartapetian et al., 2011). This review focuses on the caspase-like activities linked to ER-stress induced PCD and comments on the proteases involved.

## COMPARISON OF THE REGULATION OF ER STRESS-INDUCED PCD BETWEEN PLANT AND OTHER MODEL ORGANISMS

As hinted above, PCD is an evolutionary conserved consequence of unresolved ER stress. PCD can be induced experimentally by a prolonged treatment using an ER stress-inducing chemical. Signal transduction from ER stress to apoptosis in mammalian cells has been established, although it is not fully complete. Three ER tethered proteins: protein kinase RNA-like ER kinase (PERK), activating transcription factor (ATF6) and inositol-requiring enzyme (IRE1α) are involved in the regulation of ER stress-induced apoptosis in mammalian cells. Inside the ER lumen, the protein chaperone binding immunoglobulin protein (BiP) contributes to both refolding of misfolded proteins and activation of PERK, ATF6, and IRE1α. During ER stress, PERK activation is due to its dissociation from BiP. Active PERK phosphorylates eukaryotic initiation factor 2 (eIF2) which results in translation inhibition (Harding et al., 1999; Harding et al., 2000). However, not all protein translation is blocked and a cAMP response element-binding transcription factor ATF4 escapes the inhibition of protein translation (Harding et al., 2003). Although many ATF4 target genes are pro-survival, one of ATF4 target genes, C/EBP homolog protein (CHOP), is pro-apoptotic (Zinszner et al., 1998). The mechanism of CHOP-induced apoptosis relies on (1) Restoration of translation via dephosphorylation of eIF2 by a protein phosphatase 1 (GADD34), which is a target of CHOP; (2) Inhibition of the pro-survival serine/threonine kinase Akt; (3) Repression of anti-apoptotic protein B-cell lymphoma 2 (BCL-2) expression level. In addition to PERK, ATF6 is involved in regulating CHOP expression as the CHOP gene is a direct target of ATF6 (Yoshida et al., 2000). However, not many evidences support that ATF6 is directly involved in the apoptosis signal pathway, apart from that ATF6 is able to increase CHOP transcript level. Finally, in the IRE1α branch, activation of IRE1α is also through dissociation from BIP (Szegezdi et al., 2006). Active IRE1α is able to recruit TNF-receptor-associate factor 2 (TRAF2), which in turn recruit apoptosis-signal-regulating kinase (ASK1; Nishitoh et al., 1998). The IRE1α-TRAF2-ASK1 complex activates c-Jun N-terminal kinase (JNK). Activation of JNK activates downstream pro-apoptotic signals. For instance, JNK represses BCL-2 via phosphorylation (Davis, 2000). Downstream of PERK, ATF6, and IRE1α mediating signals is the activation of caspases. Caspase-12 (in rodent) and caspase-4 (in human) were reported to mediate a caspase cascade in ER stress-induced PCD in mammalian cells (Nakagawa et al., 2000). A recent report implicated caspase-2 in ER stress-induced PCD of mouse embryonic fibroblasts (MEFs). Under severe ER stress, IRE1α, via its α RNase activity, degrades a selective set of miRNAs which repressed caspase-2 expression level (Upton et al., 2012). Supporting the importance of IRE1α in this process, no expression of *caspase-2* and no activation of caspase-3 were observed in IRE1α knock out MEFs, and knock out of the pro-apoptotic BAX/BAK did not affect caspase-2 expression level. The discovery that caspase-2 expression level was controlled by IRE1α and not by BAX/BAK suggested a direct signal pathway from IRE1α to caspase-2 and then to the mitochondrion apoptotic pathway involving BAX/BAK (Upton et al., 2012).

When looking into the ER stress-induced PCD signal pathway in plants, many of the regulators discovered in mammalian ER stress-induced apoptosis are missing from the genome sequences or at least functional equivalent are yet to be reported. For example, a PERK homolog has not been identified in plants and Kamauchi et al. (2005) reported that, unlike in animal cells, ER stress had no effect on protein translation in *Arabidopsis*. For this the authors used S^35^ labeled methionine and cysteine to measure nascent protein expression level under ER stress in *Arabidopsis*. In addition, the phosphorylation level of eIF2α showed a decrease rather than the increase described in animal cells (Kamauchi et al., 2005). All these experiments suggested that ER stress does not induce translation attenuation in plant cells. So it is possible that PERK and its downstream PCD regulator, CHOP, do not have functional homologs in *Arabidopsis*. Besides PERK and CHOP, there is no functional homolog of the BCL-2 family members reported in plants, in particular BAX/BAK, which control mitochondria membrane permeabilization during PCD. There are therefore clear differences in the regulation of ER stress-induced PCD between plant and mammalian cells. However, some ER-stress PCD regulators are conserved between plant cells and mammalian cells such as BAX-inhibitor and it is remarkable that despite the absence of caspase homologs in plants (Watanabe and Lam, 2008) caspase-like activities are induced during ER stress-induced PCD. In particular, caspase-3-like and caspase-9-like activities are triggered in soybean suspension cells during ER stress-induced PCD activated using cyclopiazonic acid (CPA), a chemical which inhibits ER Ca^2^^+^-ATPase and causes Ca^2^^+^ efflux from the ER (Zuppini et al., 2004). Also in soybean, overexpression of N-rich proteins NRP-A and NRP-B or of a NAC transcription factor GmNAC81 (former GmNAC6), resulted in the induction of UPR and an increase of caspase-1-like and caspase-3-like activity (Costa et al., 2008; Faria et al., 2011). These few reports point out at caspases-like proteases being components of ER stress-induced PCD in plants. The next question is what are the proteases responsible for these caspase-like activities and do they regulate PCD?

## PLANT CASPASE-LIKE ACTIVITIES AND ER STRESS-INDUCED PCD

Caspase-like activities has been observed in plant during PCD consistently since their first report (del Pozo and Lam, 1998). However, it took a long period of time to identify plant proteases responsible for the caspase-like activities. Initially, plant scientists pinned their hopes on metacaspases, as metacaspases were identified as distant relatives of animal caspases (Uren et al., 2000). Indeed metacaspases share some structural features with caspases. For example, metacaspases have a catalytic dyad based on cysteine and histidine (Uren et al., 2000). It is now clearly established, however, that metacaspases do not possess caspase-like enzymatic activity at all, as their cleavage site requires an Arg in P1 (R, e.g., RR) rather than Asp (D, e.g., YVAD, DEVD; Vercammen et al.,2004; He et al., 2008; Tsiatsiani et al., 2011); Other plant proteases were found to exhibit caspase-like activities.

### CASPASE-1-LIKE ACTIVITY: VACUOLAR PROCESSING ENZYME

Vacuolar Processing Enzyme (VPE) is a plant protease localized in the vacuole and responsible for processing vacuolar proteins. Hatsugai et al. (2004) reported that VPE, in addition to its activity against the tetrapeptide ESEN, exhibited caspase-1-like enzymatic activity as shown by activity labeling using the caspase-1 inhibitor YVAD-CHO. First, protein extracts from *Nicotiana benthamiana *were labeled with biotin-x-VAD-FMK. Two bands at 40kDa and 38kDa were detected and the labeling of these two bands was reduced with the addition of the caspase-1 inhibitor ac-YVAD-CHO or of a VPE antibody (Hatsugai et al., 2004). This indicated that VPE possessed caspase-1-like activity in plants. In addition, the recombinant γVPE recognized the VPE substrate ESEN with a Km of 30.3 μm and the caspase-1 substrate YVAD with a Km of 44.2 μm (Kuroyanagi et al., 2005) Further experiments suggested that VPE participate in plant PCD via controlling tonoplast rupture (Hatsugai et al., 2004). The fact that VPE has caspase-1-like activity had been documented for almost ten years, whereas a proof that VPE regulated ER stress induced PCD was only established recently using *Piriformospora indica *and* Arabidopsis* as an experimental system. In order for the mutualistic fungi *P. indica* to successfully colonize *Arabidopsis* roots, roots need to undergo a localized PCD (Qiang et al., 2012). A hallmark of PCD during the colonization of *P. indica* was the collapse of tonoplast. In addition, VPE null mutant reduced PCD as *P. indica* colonized roots (Qiang et al., 2012). Interestingly, Qiang et al. (2012) observed a swelling of ER, which was considered as an indication of ER stress, during *P. indica* colonization. To trigger ER stress-induced PCD, *P. indica* needs to overcome UPR, which tends to dampen the ER stress response. There is evidence that this is done by repressing UPR genes expression during colonization (Qiang et al., 2012). This set of experiments implied that VPE was downstream of UPR and part of the ER-PCD pathway. A recent report brings more details to this model. Two NAC transcription factors in soybean, GmNAC30 and GmNAC81, that induce PCD downstream of osmotic and ER stress, are able to interact with each other in a synergistic manner to activate directly *VPE* gene expression (Mendes et al., 2013). Both *GmNAC30* and *GmNAC81* transcript levels increased under ER stress induced using tunicamycin (Mendes et al., 2013).

### CASPASE-3-LIKE ACTIVITY: PROTEASOME

26S proteasome is a proteases complex responsible for protein degradation in eukaryotic cells. The core unit of the 26S proteasome is the 20S proteasome. It consists of α and β subunits. Adding proteasome inhibitors, β-lactone and APnLD-CHO, to *Arabidopsis* leaf extract strongly reduced caspase-3-like activity as measured using the substrate DEVD (Hatsugai et al., 2009). In addition, the 20S proteasome β subunit 1 (PBA1) was detected by antibodies in protein-pull-down using the substrate analog inhibitor biotin-DEVD-FMK (Hatsugai et al., 2009). The activities of proteasome β subunit 2 (PBB) and 5 (PBE) were partially repressed by the caspase-3 inhibitor ac-DEVD-CHO at high concentration (>5 μM; Hatsugai et al., 2009). However, the authors suggested that neither PBB nor PBE possesses caspase-3-like activity, because the inhibition of their activities by ac-DEVD-CHO was not dose dependent (Hatsugai et al., 2009). Further to this, Han et al. (2012) purified a caspase-3-like activity from *Poplar *xylem tissues. In the elution fractions which showed highest caspase-3-like activity, the authors detected several proteins and mass spectrum analysis identified those proteins as proteasome subunit homologs in *Poplar* (Han et al., 2012). In other word, the caspase-3-like activity detected in plants is at least in part due to proteasome activity. In the context of ER stress-induced PCD, one major function of the proteasome is disposing of misfolded proteins in a process termed ER Associated Degradation (ERAD; Liu and Howell, 2010). Blocking proteasome activity is believed to reduce the degradation of misfolded proteins, which in turn causes more burdens to the ER. This implies that PBA1, as a proteasome subunit, may play a pro-survival role rather than a pro-cell death role in plant ER stress-induced PCD despite its caspase-3-like activity. So far, very few reports have depicted the role of the proteasome in PCD. Both pro-cell death and anti-cell death functions have been documented. Silencing *PBA1* blocked pathogen-induced PCD in *Arabidopsis* by blocking one of the first steps consisting of tonoplast and plasma membrane fusion (Hatsugai et al., 2009). On the other hand, silencing the 20S α6 subunit or the RPN9 subunit of 19S did elicit PCD in *N. benthamiana* (Kim et al., 2003). Similar contrasting observations have been made in animal cells. Proteasome inhibition using MG132 in human Jurkat cell increased the expression level of BIP, a marker of increased ER stress and resulted in more apoptosis (Park et al., 2011). Another report, based on vascular smooth muscle cells, also indicated that proteasome inhibition sensitized cells to ER stress-induced PCD (Amanso et al., 2011). This may be because applying both an ER stress inducer and a proteasome inhibitor may result not only in more misfolded protein aggregation, but also in a compromised UPR induction. In support of that, cells treated with the proteasome inhibitor MG132 exhibit a repressed UPR genes induction (Lee et al., 2003); how MG132 reduced UPR gene induction is not clear. On the other hand, a pro-apoptotic role of proteasome was also reported. Egger et al. (2007), reported that inhibition of the proteasome using lactacystin block ER stress-induced cell death in Rat 6 embryo fibroblasts (R6; Egger et al., 2007). The authors postulated that proteasome inhibition abolished the degradation of anti-apoptotic BCL-2 family members, so that apoptosis was blocked (Egger et al., 2007). It should be noted that a particular way of inducing ER stress was used in this research, as cells were co-incubated with brefeldin A (BFA) and cycloheximide (CHX). BFA blocks protein transport from ER to Golgi, causing ER stress. CHX represses bulk protein translation including pro-survival UPR proteins, such as proteins downstream of *ATF6* (Egger et al., 2007). With this combination of chemicals, protein input into ER was reduced and proteasome inhibition resulted only in a moderate unfolded protein burdens to ER, while BCL-2 degradation was blocked. Our lab is investigating whether the plant proteasome exhibits a pro- or an anti-cell death role in ER stress-induced PCD. Besides the proteasome, our unpublished results indicate that at least one other protease exhibit caspase-3-like activity in *Arabidopsis*.

### OTHER CASPASE-LIKE ACTIVITIES

Besides the proteases responsible for caspase-1 and caspase-3-like activities involved in ER stress induced PCD, other proteases with caspase-like activities are known in plants. Phytaspase and saspase were found to exhibit caspase-like activities (Vartapetian et al., 2011). So far, however, no report has shown the involvement of these proteases in ER stress-induced PCD. Phytaspase, a subtilisin-like serine protease, takes its name from being a plant ASPartate-specific protease and was purified from tobacco and rice. It exhibits a preferential cleavage activity against caspase-6 substrate VEID although it can cleave other caspase substrates such as caspase 1 (YVAD) and 9 (LEHD) in particular (Chichkova et al., 2010). Phytaspase was shown to mediate HR-induced PCD and ROS-induced PCD (Chichkova et al., 2010). Saspase (Serine protease with an asp cleavage site) is a subtilisin-like serine protease, purified from *Avena sativa*, that exhibits an enzymatic activity against caspase-6 substrates (VEHD, VKMD, VNLD) and one caspase-8 substrate (IETD; Coffeen and Wolpert, 2004). A role for caspase-2 and caspase-4 have been described in mammalian ER stress, but caspase-2-like or caspase-4-like activity has not been reported in plants. As caspase-2 was reported to regulate ER stress-induced apoptosis in mammalian cells (Upton et al., 2012), caspase-2 activity could be tested in plant during ER stress-induced PCD although this activity in itself does not indicate a role in plant PCD. The caspase-like activities in plants and their correlations with ER stress-induced PCD are summarized in **Table 1**.

**Table 1:** Plant caspase-like activities and ER stress-induced PCD.

Protease	Preferred caspase substrate	Reference	Species	Comments
Vacuolar processing enzyme (VPE)	YVAD, caspase-1-like	Hatsugai etal. (2004), Qiang etal. (2012), Mendes etal. (2013)	*Arabidopsis*, *soybean*, *N. benthamiana*	Controls tonoplast rupture during PCD. Positively regulate ER stress-induced PCD.
Proteasome subunit β 1 (PBA1)	DEVD, caspase-3-like	Hatsugai etal. (2009), Han etal. (2012)	*Arabidopsis*, *poplar*	Mediates PCD during HR. Activity detected during ER stress-induced PCD
Phytaspase	VEID, caspase-6-like	Chichkova etal. (2010)	*N. tabacum*, *rice*	Mediates HR and ROS-induced PCD. Not reported in ER stress-induced PCD.
Saspase	VEHD, caspase-6-like; IETD, caspase-8-like	Coffeen and Wolpert (2004)	*Oat*	Not reported in ER stress-induced PCD

## CONCLUSION

ER stress-induced PCD is gaining more and more attention in plant science research. So far, among plant proteases with caspase-like activity, only VPE, with a caspase-1-like activity, has been implicated in regulating ER stress-induced PCD. VPE is proposed to control indirectly tonoplast rupture during PCD. The detailed mechanism by which VPE controls tonoplast rupture is still unclear and could be addressed using ER-stress induced PCD as an experimental system. Another aspect that can be addressed is the role of caspase-3-like activity and of the proteasome. In particular, it is worth investigating whether in addition to being responsible for the degradation of misfolded proteins, the proteasome could also indirectly modulate UPR gene expression as suggested in some mammalian cell models (Lee et al., 2003; Amanso et al., 2011). Since three caspase-like activities have been directly or indirectly implicated in ER stress-induced PCD in plants, a complete profile of caspase-like activities during ER stress-induced PCD could be carried out using substrates and inhibitors. Following this, using relevant KO lines or KD lines would provide clearer experimental outcomes. Such an approach would give an insight into which additional proteases are involved in ER stress-induced PCD in plants. For example, whether phytaspase, a PCD regulator, has a function in ER stress-induced PCD is an interesting question that is still open. Finally, a complete understanding will not be achieved without knowledge of the *in vivo* protein substrates implicated.

## Conflict of Interest Statement

The authors declare that the research was conducted in the absence of any commercial or financial relationships that could be construed as a potential conflict of interest.
